# Pulmonary disease in diabetes

**DOI:** 10.1111/1753-0407.13509

**Published:** 2023-12-29

**Authors:** Zachary Bloomgarden

**Affiliations:** ^1^ Department of Medicine, Division of Endocrinology Diabetes and Bone Disease, Icahn School of Medicine at Mount Sinai, New York New York USA

Diabetes is associated with a variety of pulmonary disorders beyond its associations with sleep apnea discussed in the previous commentary. The Fremantle Diabetes Study in Western Australia showed that persons with type 2 diabetes (T2D) had 5%–12% reduction in vital capacity and expiratory flow rates with evidence of worsening over a 7‐year period of observation; in follow‐up, each 10% reduction in the forced expiratory volume in 1 s (FEV1) was associated with a 13% increase in mortality controlling for age, sex, duration, hypertension, retinopathy, neuropathy, albuminuria, and coronary disease.^1^ This relationship extends to prediabetes. Among 2332 initially nondiabetic persons in Malmö, Sweden, homeostatic model assessment of insulin resistance (HOMA‐IR) was elevated at 10‐year follow‐up in approximately one third of those in the lowest quartile of baseline forced vital capacity (FVC), and each 10% greater FVC was associated with a 10% lower likelihood of IR and with 10% lower likelihood of diabetes, adjusted for age, smoking, and body mass index (BMI), while cardiovascular disease (CVD) events significantly increased among those persons with FVC below the median who had developed insulin resistance.^2^ Meta‐analyses of 39 studies of 1274 persons with T1D and 1353 controls and of 66 studies of 11 134 persons with T2D and 48 377 controls both showed 6%–10% lower pulmonary flow rates than those among controls.^3^, ^4^ The UK Biobank Study compared 372 093 nondiabetic persons (glycosylated hemoglobin [HbA1c] < 5.7), 53 378 with prediabetes (HbA1c 5.7–6.4), and 27 209 with diabetes (HbA1c ≥ 6.5), adjusting for factors including age, sex, ethnicity, BMI, education, and cigarette and alcohol use; those with prediabetes and diabetes had a 10‐year chronic obstructive pulmonary disease (COPD) relative risk of 1.18 (1.13–1.24) and 1.35 (1.24–1.47), respectively, with preexisting diabetes associated with a ~1.2‐fold greater risk than that among those diagnosed at the time of study entry, and with COPD‐specific mortality nearly doubled among those with diabetes diagnosed ≥7 years prior to study entry.^5^ In a study of 48 nondiabetic persons, 68 with prediabetes, 29 newly diagnosed T2D, and 110 with long‐term T2D, symptoms of breathlessness were present in none, 3%, 10%, and 15% of the respective groups, along with reduction in FVC and pulmonary diffusing capacity.^6^

It is, then, to be expected that a variety of lung diseases occur in association with diabetes. Asthma prevalence is more than doubled with diabetes, with greater severity and with evidence of airway inflammation and hyperresponsiveness. COPD also shows greater severity among people with diabetes, in part related to obesity and to systemic inflammation. People with idiopathic pulmonary fibrosis (IPF) and with pulmonary hypertension (PH) are more likely to have diabetes, and although it is not clear that their prevalences are greater among people having diabetes, there is evidence that diabetes is associated with greater severity of these conditions as well.^7^ COPD is associated with many risk mediators in common with those of diabetes, including cigarette use, aging, hypertension, dyslipidemia, and insulin resistance, and there is evidence that COPD is associated with low‐grade systemic inflammation and to hypercoagulability, and that exacerbations of COPD may be related to heart failure and to coronary heart disease, with elevation in troponin and in basic natriuretic peptide (BNP) often accompanying such episodes.^8^ A meta‐analysis comparing 25 180 persons with IPF to 73 434 controls showed the likelihood of diabetes to be 1.54‐fold greater in the former group, although evidence of diabetes as a risk factor for IPF was not found.^9^ Part of the difficulty may be in underdiagnosis, with a study of 53 persons with T2D and unexplained exertional dyspnea showing lower peak oxygen uptake during exercise and higher mean pulmonary artery pressure.^10^ The association of IPF severity with diabetes may be related to oxidative stress, to endothelial dysfunction, to inflammation, to obesity, to diaphragmatic muscle dysfunction, to autonomic neuropathy, and to albuminuria.^11^, ^12^ Insulin resistance has been proposed as underlying the relationship between diabetes and PH, with consequent inflammation, dyslipidemia, and endothelial dysfunction leading to adverse pulmonary vascular remodeling and to right ventricular dysfunction.^13^ In the Fremantle Diabetes Study, analysis of the subset of persons with T2D who had echocardiograms obtained for clinical reasons showed 6.4% and 2.6% prevalence of estimated right ventricular systolic pressure >30 and >40, respectively, and over 6.6‐year follow‐up an additional 9.2% and 5.0% developed this degree of PH, suggesting that the risk of PH is approximately 40% greater among persons with diabetes.^14^ A study of patients with PH having versus not having diabetes showed that the former had higher BNP, shorter 6‐min walking distance, more dyspnea, higher pulmonary artery pressure, and shorter survival,^15^ and a study of 110 563 persons with newly diagnosed PH in the US national Veterans Health Affairs database showed that more than one third had diabetes, which was associated with a 1.21‐fold greater mortality risk.^16^ Genetic association studies in a population of 14 861 persons with echocardiogram‐measured pulmonary pressure and right ventricular function suggest both T2D and obesity to be associated with greater levels of tricuspid regurgitation and right ventricular systolic pressure, suggesting both to play roles in the development of PH.^17^

A number of approaches to diabetes treatment may have pulmonary benefit. It should be noted that epidemiologic study findings need to be regarded with skepticism, a caution which was recently raised in analysis of potential biases affecting the evidence that metformin may lead to reduction in cancer and/or to improved outcome of persons being treated for cancer.^18^ Bearing in mind this caveat, we can review several interesting reports. In a study of 3599 adults with both IPF and T2D, controlling for age, sex, race/ethnicity, residence region, year, medications, oxygen use, smoking status, healthcare use, and comorbidities, those treated with metformin had 54% lower mortality and 18% lower risk of hospitalization.^19^ In a study of 350 536 persons with new‐onset diabetes not having COPD, pioglitazone treatment was associated with lower likelihood of development of COPD, particularly among those with longer duration of pioglitazone treatment.^20^ Thiazolidinediones are, however, complex agents to administer to people with cardiac and/or pulmonary disease, with a study of hospitalizations among 402 153 persons with both T2D and COPD showing those treated with a thiazolidinedione having >50% increase in likelihood of CVD and of heart failure events, as well as increased risks of development of bacterial pneumonia, of requirement for noninvasive positive pressure ventilation during hospitalization, and of development of lung cancer.^21^

In the monocrotaline‐treated rat PH model, the sodium‐glucose co‐transporter‐2 inhibitor (SGLT2i) empagliflozin reduced right ventricular and pulmonary artery pressures, decreased right ventricular hypertrophy and fibrosis, and was associated with improved survival.^22^ A 12‐week trial of 65 persons with heart failure and an implanted pulmonary artery pressure sensor revealed that those randomized to receive empagliflozin had a reduction in pulmonary artery diastolic pressure.^23^ In an epidemiologic study of persons with diabetes and COPD using the UK Clinical Practice Research dataset, those treated with a SGLT2i had a 41% reduction in COPD exacerbations requiring hospitalization in comparison to those treated with a sulfonylurea, although moderate exacerbations treated on an outpatient basis were not more common.^24^

The greatest degree of improvement appears to be seen with the use of glucagon‐like peptide 1 receptor agonists (GLP‐1RA). GLP‐1 receptor expression in the lungs is greater than that in most other tissues, and administration of GLP‐1RA reduces the pulmonary response to inhaled allergens in a mouse model and decreases bronchial hyperresponsiveness and inflammatory markers in animal models.^25^ In the epidemiologic study using the UK Clinical Practice Research dataset, severe and moderate COPD exacerbations were reduced by 41% and by 48%, respectively.^24^ A similar study from the Mass General Brigham dataset showed the greatest reduction in both severe and moderate COPD exacerbations with GLP‐1RA treatment, with SGLT2i appearing to be associated with benefit as well.^26^ A third study compared the risk of chronic lower respiratory disease exacerbations with GLP‐1RA versus dipeptidyl peptidase IV inhibitor (DPP‐IVi) in 4150 and 12 540 persons, respectively, finding a 48% reduction with GLP‐1RA in likelihood of hospitalization and a 30% reduction in total pulmonary exacerbations.^27^ A meta‐analysis of 28 randomized controlled trials of GLP‐1RA involving 77 485 participants showed a 14% decrease in likelihood of overall respiratory disease with a 34% lower likelihood of pulmonary edema and a 14% lower likelihood of bronchitis.^28^

In summary, there is convincing evidence that diabetes is associated with a decrease in pulmonary function. A variety of mechanisms appear to be involved, including insulin resistance, endothelial dysfunction, inflammation, and effects of obesity. PH, pulmonary fibrosis, asthma, and COPD all have adverse associations with diabetes. A variety of diabetes treatment approaches appear of benefit in treating these pulmonary conditions, with experimental evidence in animal studies and randomized controlled trials of benefit of SGLT2i, and with strong epidemiologic evidence of benefit of the GLP‐1RA class.
